# Population Pharmacodynamic Modelling of the CD19+ Suppression Effects of Rituximab in Paediatric Patients with Neurological and Autoimmune Diseases

**DOI:** 10.3390/pharmaceutics15112534

**Published:** 2023-10-26

**Authors:** Natalia Riva, Lucas Brstilo, Aymara Sancho-Araiz, Manuel Molina, Andrea Savransky, Georgina Roffé, Marianela Sanz, Silvia Tenembaum, Maria M. Katsicas, Iñaki F. Trocóniz, Paula Schaiquevich

**Affiliations:** 1 Pharmacometrics & Systems Pharmacology Research Unit, Department of Pharmaceutical Sciences, School of Pharmacy and Nutrition, University of Navarra, 31008 Pamplona, Spain; aaraizsanch@alumni.unav.es (A.S.-A.); itroconiz@unav.es (I.F.T.); 2 Unit of Innovative Treatments, Hospital de Pediatría JP Garrahan, Buenos Aires C1245 CABA, Argentina; brstilolucas@gmail.com (L.B.); molinamanuel40@gmail.com (M.M.); paula.schaiquevich@gmail.com (P.S.); 3 National Council of Scientific and Technical Research (CONICET), Buenos Aires C1425 FQB, Argentina; 4 Navarra Institute for Health Research (IdiSNA), 31008 Pamplona, Spain; 5 Neurology Service, Hospital de Pediatría JP Garrahan, Buenos Aires C1245 CABA, Argentina; andreagsavransky@gmail.com (A.S.); silviatenembaum@gmail.com (S.T.); 6 Laboratory of Cellular Immunology, Hospital de Pediatría JP Garrahan, Buenos Aires C1245 CABA, Argentina; georro87@gmail.com (G.R.); marianelasanz@hotmail.com (M.S.); 7 Immunology and Rheumatology Service, Hospital de Pediatría JP Garrahan, Buenos Aires C1245 CABA, Argentina; mmkatsi@yahoo.com.ar; 8 Institute of Data Science and Artificial Intelligence, DATAI, University of Navarra, 31009 Pamplona, Spain

**Keywords:** paediatrics, rituximab, NONMEM, biosimilar pharmaceuticals, pharmacodynamics

## Abstract

Background: Limited pharmacotherapy and the failure of conventional treatments in complex pathologies in children lead to increased off-label use of rituximab. We aimed to characterize the time course of CD19+ B lymphocytes (CD19+) under treatment with intravenous rituximab in children with neurologic and autoimmune diseases and to evaluate the impact of covariates (i.e., demographics, diagnosis and substitution between innovator and biosimilar product) on rituximab pharmacodynamics and disease activity. Methods: Pre- and post-drug infusion CD19+ in peripheral blood were prospectively registered. A population pharmacodynamic model describing the time course of CD19+ was developed with NONMEM v7.4. Simulations of three different rituximab regimens were performed to assess the impact on CD19+. Logistic regression analysis was performed to identify predictors of clinical response recorded through disease activity scores. Results: 281 measurements of CD19+ lymphocyte counts obtained from 63 children with neurologic (*n* = 36) and autoimmune (*n* = 27) diseases were available. The time course of CD19+ was described with a turn-over model in which the balance between synthesis and degradation rates is disrupted by rituximab, increasing the latter process. The model predicts half-lives (percent coefficient of variation, CV(%)) of rituximab and CD19+ of 11.6 days (17%) and 173.3 days (22%), respectively. No statistically significant effect was found between any of the studied covariates and model parameters (*p* > 0.05). Simulations of different regimens showed no clinically significant differences in terms of CD19+ repopulation times. A trend towards a lack of clinical response was observed in patients with lower CD19+ repopulation times and higher areas under the CD19+ versus time curve. Conclusions: Rituximab pharmacodynamics was described in a real-world setting in children suffering from autoimmune and neurologic diseases. Diagnosis, substitution between innovator rituximab and its biosimilars or type of regimen did not affect rituximab-induced depletion of CD19+ nor the clinical response in this cohort of patients. According to this study, rituximab frequency and dosage may be chosen based on clinical convenience or safety reasons without affecting CD19+ repopulation times. Further studies in larger populations are required to confirm these results.

## 1. Introduction

Rituximab is a chimeric monoclonal antibody directed against the B cell antigen CD20, a protein that is expressed on the surface of all mature B cells. Rituximab causes a transient and selective depletion of normal and malignant CD20+ B cell subpopulations and offers a targeted approach to disorders caused by B cells [1]. Noticeably, rituximab has the approval for its use in adults with oncological and autoimmune pathologies, but it is unlicensed for most paediatric diseases due to a lack of systematic clinical evidence [2] leading to a large number of off-label indications in this population. Nonetheless, off-label use of rituximab has been accepted worldwide in protocols and has scientific consensus in multiple clinical scenarios [3,4].

Frequently in daily practice, the efficacy of rituximab is evaluated through the (i) depletion and repopulation rate of peripheral CD19+ B-cells as a marker of the pharmacologic outcome of treatment with anti-CD20 therapies [5,6,7], and (ii) through the assessment of validated questionnaires of disease activity as surrogates for clinical efficacy [8,9,10,11,12]. In terms of safety, close monitoring of administrations is a common practice as infusion-related reactions are the main adverse event. These adverse drug reactions potentially affect rituximab efficacy through IgG antidrug antibody production, increasing drug clearance [13].

In recent years, the expiration of the patent of the innovative product has led to the introduction of biosimilars of rituximab approved by the European Medicines Agency (EMA) and the Food and Drug Administration (FDA). Approval of biosimilars is supported by extensive analytical and preclinical studies, while pharmacokinetics (PK), efficacy and safety data mostly result from adult patient evaluations with scarce studies performed in the paediatric population [14,15,16]. Although interchangeability is not automatically granted upon biosimilar approval, in healthcare systems with limited resources, innovative and biosimilar are spontaneously substituted.

Despite previous reports have been published on the safety assessment of interchangeability of biosimilar and innovator rituximab [17], information on pharmacological and clinical efficacy upon substitution is still scarce for the paediatric population [18].

Thus, in the present study, we aimed to explore the following aspects: (1) to characterize the time-course of CD19+ B cell counts, (2) to assess the effect of biosimilar products on the pharmacodynamics of rituximab, (3) to explore the impact of different therapeutic regimens on CD19+ B cell repopulation times, (4) to evaluate the clinical response through disease activity scores with rituximab treatment in paediatric patients with autoimmune and neurologic diseases treated with rituximab, and (5) to identify predictors of clinical response with special emphasis on the relation with CD19+ lymphocyte count.

## 2. Materials and Methods

### 2.1. Study Design, Patients, and Drug Administration

This work represents a prospective study, analysing patients treated with rituximab between January 2019 and December 2021 at Hospital de Pediatria JP Garrahan, Buenos Aires, Argentina. The Institutional Research Ethics Committee (protocol #1315) approved the study and parents and/or guardians gave their informed consent.

Inclusion criteria consisted of paediatric patients with neurologic, immunologic, hematologic and rheumatologic (IHR) diseases, who were eligible by the clinical team to receive rituximab based on failure of previous treatments and whose medical records included CD19+ B lymphocytes assessment before and after rituximab infusion. Exclusion criteria were applied for patients subjected to plasmapheresis.

Patients received a complete cycle of intravenous rituximab in continuous infusion over approximately 6 h at a dose of 375 mg/m^2^ once weekly for 4 weeks, or 2 doses of 500–750 mg/m^2^ every 15 days. Institutionally, re-administration of a new cycle or infusion of rituximab after completion of the first cycle is considered by the treating physician based on the disease progression and repopulation of CD19+ B lymphocytes, which occurs approximately at 6 months post-cycle [19,20].

As mentioned, rituximab treated patients in the study period were prospectively followed-up, including patients that received their first cycle during this period as well as those that received second cycles. It is expected that patients change their disease status after rituximab treatment, and this may affect the PK and/or the PD of the drug. Therefore, we grouped patients in the model building cohort if they had data of the first cycle of rituximab treatment and in the external predictive evaluation cohort for those that had data on subsequent cycles.

The following information was collected from the medical records during the stay at the hospital: diagnosis, age, sex, body weight, body surface area (BSA), CD19+ B lymphocytes (%) and absolute lymphocyte values to calculate the absolute value of CD19+ B lymphocytes. Liver and kidney function tests (alanine aminotransferase (ALT), aspartate aminotransferase (AST), γ-glutamyl transferase (GGT), albumin and total bilirubin, serum creatinine) were registered. Collected information related to rituximab infusion included dose, infusion rate, and trademark (innovator Mabthera^®^ Roche, Mannheim, Germany or biosimilar Novex^®^, Elea, Buenos Aires, Argentina). Concomitant drugs potentially affecting CD19+ counts were registered including steroids, cyclophosphamide, methotrexate, and intravenous immunoglobulin.

### 2.2. Rituximab Pharmacologic Response: Blood Sampling and Flow Cytometry

Blood samples for routine CD19+ measurements were drawn before starting rituximab treatment and 1 to 3 months or every 6 months after rituximab infusion, based on patient clinical evolution and critical conditions. Determination of CD19+ B lymphocytes was performed using flow cytometry with a BD FACSCalibur™ analyser (San Jose, CA, USA), in peripheral blood samples with a limit of quantification (LOQ) of 10 × 10^6^ cells/L.

### 2.3. Population Kinetic-Pharmacodynamic Analysis

CD19+ profiles were analysed based on the population approach with the Laplacian estimation method [21]. Data were logarithmically transformed for the analysis. Inter-individual variability (IIV) was modelled exponentially. Residual variability was described by selecting the most appropriate residual model structure among the additive, proportional, or combined error models. CD19+ values reported as below the limit of quantification (BLQ) were also considered in the analysis and treated as censored information with the M3 method [22].

During the process of model building, selection between model candidates was based on biological plausibility, parameter precision, visual inspection of the goodness of fit plots and the change in the value of the minimum objective function which approximates to −2xlog (likelihood) [−2LL]. For two nested models differing in one parameter, reductions in −2LL of 3.84 and 6.635 points were considered statistically significant at the 0.05 and 0.01 levels of significance, respectively.

Population pharmacodynamic analysis was performed using the software NONMEM 7.4 and Pearl speaks NONMEM software [23,24,25]. Model management and stepwise covariate model building were carried out through PsN using Pirana 2.9.9 [26]. Dataset pre-processing and figures were performed with R 4.0.2 (R Foundation for Statistical Computing, 2017), and RStudio 1.3.1073 (RStudio Team, 2020).

The analysis followed three steps: (1) development of the base population model, (2) covariate selection, and (3) model evaluation, external predictive performance evaluation, and exploration through simulations.

#### 2.3.1. Base Population Model

A model describing adequately the data obtained after the first administration cycle (195 CD19+ observations, 52 patients) without the inclusion of any covariates, was developed at this stage.

Equation (1) provides the mathematical representation of the model structure that describes the CD19+ profiles in the absence and presence of rituximab (RTX).

(1) $$\frac{dCD19+}{dt}=K_{syn}-K_{deg}\times \left[1+S\left(t\right)\right]\times CD19+$$

where $\frac{dCD19+}{dt}$ represents the rate of change of CD19+, and K_syn_ and K_deg_ are the zero and first-order rate constants of synthesis and degradation, respectively, governing the turn-over mechanisms of CD19+ in peripheral blood. S(t) refers to the CD19+ depletion effects of rituximab. At steady-state conditions, ($\frac{dCD19+}{dt}=0$), CD19+_0_ is the CD19+ level at baseline and equals to K_syn_/K_deg_.

Concerning the random effects, the structure (diagonal and off-diagonal elements) of the Ω variance-covariance matrix was investigated as well as the best model describing the residual error.

#### 2.3.2. Covariate Selection

Exploratory data analysis was performed to evaluate correlations between continuous covariates and to check the homogeneous distribution of categorical covariates. Missing data were handled with the mean imputation approach. We defined a variable named “switch” that took the value of 0 if the patient received the same trademark during the entire cycle or 1 if the innovator and the biosimilar were infused at least once during the cycle. The effect of trademark substitution on rituximab pharmacodynamics was also investigated by considering a time-dependent categorical covariate named as “brand” assigning the last trademark received to the CD19+ values obtained. Covariates were tested in all parameters according to biological plausibility and selection was performed through the stepwise covariate modelling approach. The 0.05 and 0.01 levels of significance during the forward inclusion and backward deletion procedures were used, respectively [27,28].

#### 2.3.3. Model Evaluation, External Predictive Performance Evaluation and Exploration

The simulation-based diagnostic prediction-corrected visual predictive checks (pc-VPC) was used to evaluate model performance [29]. Five hundred datasets of the same characteristics as the original were simulated using the selected model and the corresponding parameter estimates. For each simulated dataset and sampling bin, the 2.5th, 50th and 97.5th percentiles of the simulated CD19+ were calculated. Then, the area corresponding to the 95% prediction interval of each percentile was computed and represented graphically together with the 2.5, 50th and 97.5th percentiles of raw data. In addition, for the data below the quantification limit (LLOQ), the proportion of LLOQ and the 95th prediction interval of predicted data were computed. To further evaluate the reliability of the model, 500 bootstrap datasets were generated and then the mean and 95% confidence intervals of the estimates were compared with the final model estimates obtained with NONMEM. Bias and precision between individual predictions and observed CD19+ B cell counts were calculated according to references [30,31]. External predictive performance was evaluated using data from two subgroups of patients in whom CD19+ was measured beyond the first cycle of treatment. Firstly, CD19+ observations in the second cycle of a subset of seven patients, that also had available CD19+ values on the first cycle and were included in the model-building cohort, were compared to the predictions obtained using their individual parameters estimates from the first cycle. In addition, we assessed the predictive performance of the model in 11 patients that were not included in the model-building dataset because they only had available data from second cycles. In this subgroup we visually assessed the predictive performance of the model with respect to the observed data obtained by means of pc-VPC.

Finally, different rituximab treatment regimens were explored to assess the effect on CD19+ B cell repopulation time (T_CD19+_), defined as the time interval in which CD19+ levels remain below the limit of quantification after rituximab administration. T_CD19+_ was explored by simulating daily CD19+ levels during one year of treatment in five hundred virtual patients with body surface area (BSA, as rituximab is BSA-dosed in daily practice) of 1.3 m^2^ (median value of our population) under the following dosing schedules: (i) Four doses of 375 mg/m^2^ given weekly, (ii) and (iii) two doses of 500 or 750 mg/m^2^ administered every two weeks, respectively. Simulations were performed using Simulx^®^ (Lixoft SAS: Antony, France, 2019).

### 2.4. Clinical Response and Predictive Factors

Treatment response was defined in conjunction with the treating physician based on the Systemic Lupus Erythematosus Disease Activity Index (SLEDAI) clinical scores for patients with systemic lupus erythematosus [10,11], the Expanded Disability Status Scale (EDSS) for patients with neurological diseases [8,9], and Myasthenia Gravis Activities of Daily Living Scale scores [12].

Four categories of clinical responses were defined comparing the clinical scores before and after rituximab treatment as follows: complete Response (CR), final score equal to 0; partial Response (PR), the final score is lower than the basal score, with or without new symptomatology; stable disease (SD), final and basal scores are the same; and nonresponse (NR), the final score is higher than basal score. In all cases, the basal score was measured before rituximab administration. Further grouping included “unsatisfactory response” for NR and SD categories and “satisfactory response” for PR and CR.

Logistic regression was used to evaluate whether the probability of unsatisfactory response was related to one or more of the following predictors: T_CD19+_, the area under the curve of CD19+ B cell count versus time over 180 days after initiation of the first cycle of treatment (AUC_0-180_), demographics, switching brands of rituximab and co-medications. Factors significant at a *p* value of 0.2 in the univariate analysis, clinically relevant and with biological plausibility were tested in a multivariate logistic regression model. Logistic regression was performed with R 4.0.2 (R Foundation for Statistical Computing, 2017), and RStudio 1.3.1073 (RStudio Team, 2020).

## 3. Results

Patients receiving rituximab in the studied period were initially identified (*n* = 99). After the exclusion of those patients with incomplete medical records/laboratory examinations (*n* = 36), a total of 63 patients were finally included in the training cohort (*n* = 52) and external predictive performance evaluation dataset (*n* = 11), as shown in Table 1. We registered 180 infusions of rituximab with a median (range) administered dose of 500 mg (41.5–1000) and collected 281 measurements of CD19+ lymphocyte counts (52% reported as BLQ) from patients with neurologic diseases (*n* = 36) and immune-haemato-rheumatologic (IHR) diseases (*n* = 27). Concerning the neurologic diseases, the most frequent pathologies were neuromyelitis optica (*n* = 8), multiple sclerosis (*n* = 6) and immune-mediated encephalitis (*n* = 5), whereas in the IHR group, the most frequent disease was systemic lupus erythematosus (*n* = 14). The remaining pathologies are listed in Table S1.

### 3.1. Population Pharmacodynamic Modelling

A model describing adequately the data (195 CD19+ observations, 52 patients) was developed.

In the absence of rituximab, the term S(t) in Equation (1) has a value of 0.

S(t) has the following form (Equation (2))

(2) $$S(t)=\frac{E_{MAX}\times A_{RTX}}{{ED}_{50}+A_{RTX}}$$

where E_MAX_ is the maximum fold increase in K_deg_ that rituximab can elicit, and ED_50_ is the predicted amount of rituximab after its administration (A_RTX_) required to achieve 50% of the E_MAX_. During model building, additional expressions for S(t) were also evaluated such as the sigmoidal E_MAX_ and linear models.

Since concentrations of rituximab were not measured in the current study, A_RTX_ values were predicted considering the rate (K_0_) and duration of the intravenous infusions (T_inf_) received by the patients and assuming a mono-exponential decay of rituximab in blood as reflected by Equation (3):

(3) $$\frac{dA_{RTX}}{dt}=K_{0}-K_{E}\times A_{RTX}$$

The value of K_0_ equals 0 at times > T_inf_. K_E_ represents a first-order rate constant of removal of rituximab from the blood compartment. Models including a time or CD19+ dependent elimination of A_RTX_ were also considered.

During the development of the base model, results indicated that the parameter accounting for the rate of elimination of rituximab, K_E_, was not significantly affected either by the time after the first administration or the predicted systemic CD19+ levels (*p* > 0.05). Reducing Equation 2 to a linear relationship between K_deg_ and A_RTX_ significantly worsened the fit and, including sigmoidicity, did not lead to any improvement in the description of the data (*p* > 0.05). Variability was incorporated into the model for the parameters CD19+_0_, K_deg_, and K_E_, as this resulted in an improved description of the data; however, inclusion of variability in any of the pharmacodynamics-related parameters did not improve the fit. The covariance across the diagonal elements of the Ω variance–covariance matrix was non-significant (*p* > 0.05). Residual variability was best described with an additive error model in the logarithmic scale.

The tested covariates did not show significant effects on any of the parameters of the model (*p* > 0.05). Remarkably, the pathology or substitution between innovator and biosimilars (trademark was missing in 14% of the infusions) did not influence the drug-related parameter K_E_, the pharmacodynamics-related parameters or the CD19+ physiology-dependent parameters (*p* > 0.05).

**Figure 1:**
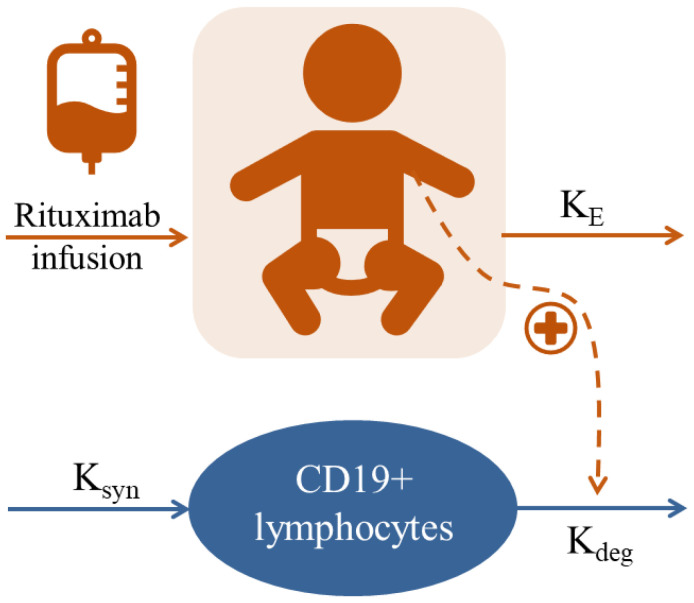
Schematic representation of the pharmacodynamic model describing the CD19+ depletion effects of rituximab. K_E_, first-order rate constant of elimination; K_syn_ zero-order constant of CD19+ synthesis; K_deg_, first-order rate constant of CD19+ elimination.

The schematic representation of the model is depicted in Figure 1, whereas the estimates of the parameters of the selected population model are listed in Table 2. Precision was in general high for all parameters. Despite the percentage relative error [RSE(%)] of the ED_50_ being higher than 50% (67%), the results for the bootstrap analysis indicated that the parameter values (SE) obtained with bootstrap were similar to the estimates obtained with NONMEM and the 95% confidence interval did not include the value of zero (Table 2).

**Figure 2:**
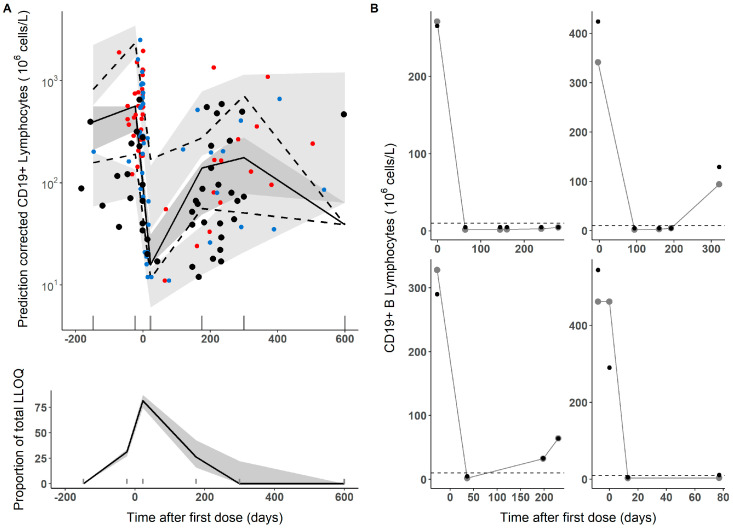
(**A**) Upper panel. Prediction-corrected visual predictive checks. The grey areas cover the 95% confidence intervals of the 2.5th, 50th and 97.5th percentiles calculated from the 500 simulated datasets. Lines represent the median (solid) and the 2.5th and 97.5th percentiles (dashed) of the raw data of the model-building dataset. Solid coloured circles show the data used to develop the model (red, neurologic diseases, blue, immune-haemato-rheumatologic diseases). Black circles correspond to the raw data from the cohort of patients used for external predictive performance evaluation (*n* = 11). Lower panel. The solid line represents the observed percentage of lower than the limit of quantification (LLOQ or below the limit of quantification, BLQ) values. The grey area covers the 95% confidence intervals of the simulations. Sticks on the x axis correspond to bins: (−147, −23, 22.5, 175, 300, and 600 days), (**B**) individual profiles of observed CD19+ cells (black points) and model predictions (grey lines and points) of four representative patients.

Figure 2A represents the results of the model evaluation using the simulation-based diagnostics pcVPC. The median tendency and the dispersion of the raw data used to develop the model appear well described by the population model (upper panel). Similarly, the percentage of samples reported as BLQ is well captured throughout the entire course of the study (lower panel). Figure 2B shows the individual observed and predicted CD19+ vs. time profiles for four representative patients taking into account four different pathologies (i.e., neuromyelitis optica, Myasthenia gravis, multiple sclerosis and systemic lupus erythematosus). Bias was −20.61% (−31.1–−10) and root mean squared error (RMSE, precision) was 52.4% (44.2–60.5).

**Figure 3:**
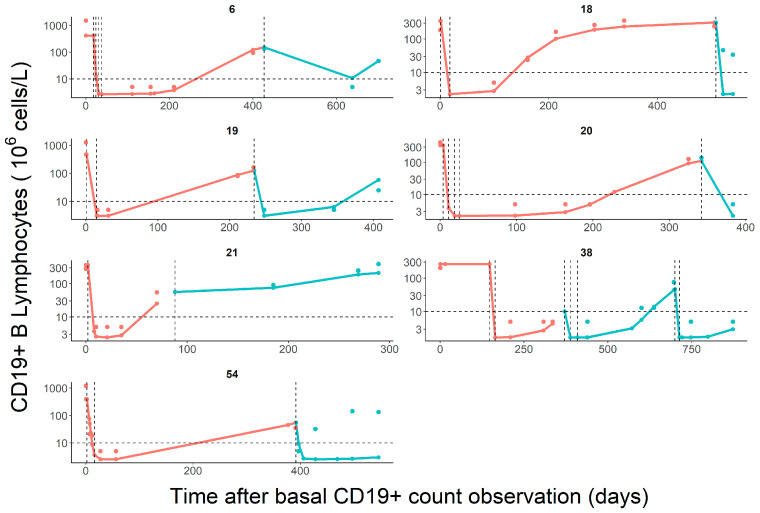
Individual predictions and simulations of the CD19+ B cell kinetics in a subgroup of patients (*n* = 7) in the first (pink lines) and in the second cycle of rituximab (blue lines), respectively. Individual observations are represented as circles. The horizontal dashed line represents the limit of quantification of CD19+ cells (10 × 10^6^ cells/L). Vertical dashed lines represent the rituximab infusions.

To evaluate the predictive performance of the model, the CD19+ profiles after the second administration were predicted in seven patients using their individual parameter estimates obtained from the first cycle. As shown in Figure 3, the corresponding model parameters obtained from the first cycle described well the dynamics of CD19+ after the second cycle of administration with special mention to ID 54 (see Section 4). In addition, the population model adequately captured the data obtained from eleven patients after repeated administration of rituximab (black circles, Figure 2A).

**Figure 4:**
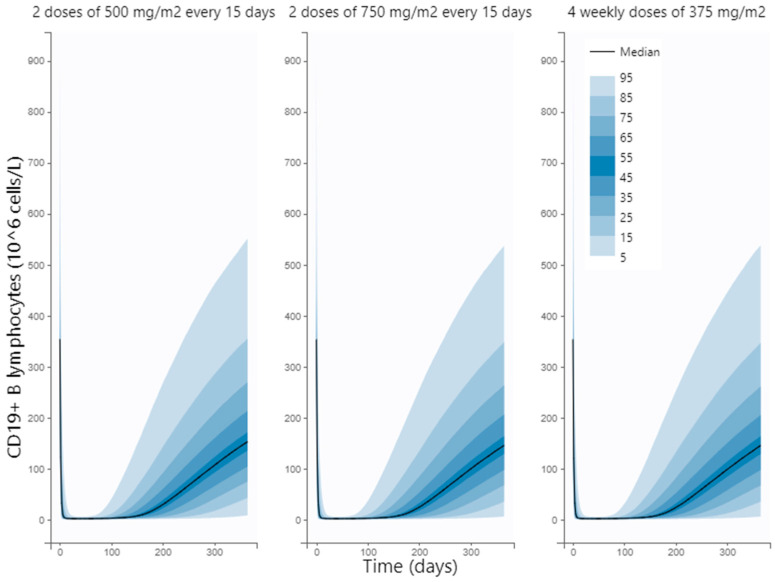
Simulated profiles of CD19+ B lymphocytes under three schemes frequently used in the clinics. Solid-black lines represent the median simulated values. Percentile bands of simulated data are represented in different shades of blue (level 90%, 9 bands).

To assess the most convenient dosing strategy in terms of CD19+ B cell count repopulation and clinical management, we explored different schemes of rituximab treatment. Interestingly, simulations of the CD19+ profiles after 4 weekly doses of 375 mg/m^2^ or two doses of 500 or 750 mg/m^2^ administered every 2 weeks revealed that the time required to achieve full CD19+ repopulation ranged from 10 to 67 weeks with a median value of 24 weeks regardless of the dosing scheme (Figure 4).

### 3.2. Clinical Response Analysis

The clinical response analysis was performed using data obtained from a subgroup of 26 patients, whose characteristics are listed in Table S2. Relevant metrics such as the AUC_0-180_ and T_CD19+_ were calculated from the simulated CD19+ profiles using the individual pharmacodynamic parameters (Figure S1). The results from the univariate analysis are shown in Table S3 and indicate that none of the explored predictors were significantly associated with the probability of clinical response (*p* > 0.05). Nevertheless, we observed a trend between lower AUC_0-180_ or higher T_CD19+_ and satisfactory response (complete or partial response, Figure S2).

Regarding the relationship between clinical response and treatment regimens (4 × 375 mg/m^2^, 2 × 500 mg/m^2^ or 2 × 750 mg/m^2^), no significant associations were observed. Nonetheless, a trend towards a lower non-response frequency (12%) was observed for the 2 × 500 mg/m^2^ scheme in comparison with the 4 × 375 mg/m^2^ or 2 × 750 mg/m^2^ schemes that resulted in a frequency of 43% of non-responders (*p* > 0.05).

## 4. Discussion

In this report, we studied the off-label use of rituximab and its biosimilar in paediatric patients with neurologic or autoimmune complex diseases and developed a population model, which describes the full CD19+ profiles during the first cycle of rituximab administration. In addition, a link between clinical efficacy (based on clinical scores) and some derived metrics of CD19+ response was attempted. None of the covariates explored (i.e., diagnosis, and substitution between innovator and a biosimilar formulation) showed a significant impact neither on the parameters of the population model nor on the clinical response.

The developed model adequately described the data and presented adequate precision in estimated parameters, with lower precision in ED50 (RSE%: 61), required to describe exposure–response relationship. The studied dose-range and the highly efficacious doses may have contributed to hampering a precise estimation.

No covariates were retained in the final model contrary to Pan et al., the only previous study in paediatrics with autoimmune diseases [18], which identified an effect of comedication on CD19+ depletion. The lack of impact in the time course of CD19+ of the co-administration of cyclophosphamide or methotrexate might be explained by the fact that, in our study, only a reduced number of patients (*n* = 7) received those two drugs simultaneously.

Although rituximab concentration levels were not available in our study, using the KPD approach allowed the estimation of the rituximab K_E_ parameter which is associated with an apparent elimination half-life of 11.6 days (95% CI: 7.7–14 days). This value is lower than the reported in paediatric patients with autoimmune diseases (19 days) [18], nephrotic syndrome (20 days) [32] and adults with rheumatoid arthritis, ANCA-associated vasculitis and nephropathies (17–23 days) [33,34,35,36], but higher than that reported for the first time for a biosimilar formulation of rituximab in paediatric patients with rheumatological conditions [37]. Among the reasons for the different half-life (or estimate of K_E_) obtained in the current evaluation, the presence of anti-drug antibodies (ADAs) represents a plausible explanation. Even though we were not able to measure ADAs, 42.3% of our patients developed infusion-related reactions [17], which may contribute to the development of ADAs as suggested by Oomen et al. [38], who showed that ADAs-positive children had a higher frequency of infusion-related adverse drug reactions, lower frequency of B cell depletion, and undetectable rituximab levels due to a higher clearance or K_E_. Thus, measurement of ADAs would be required in future studies to contribute to the understanding of associations of ADA presence and quantity with rituximab efficacy and toxicity. In addition, differences in unspecific internalization of rituximab associated with a disease-related variability in B cell subpopulation found in a recent report in SLE patients [39] may also explain the discrepancy between our results and the ones previously reported

Additional differences were found when comparing the current PKPD parameters with those reported by Pan et al. For example, our estimates of CD19+_0_ and E_MAX_ were higher (353 vs. 266 10^6^ cells/L and 155 vs. 35.2, respectively), whereas K_deg_ was lower (4 × 10^−3^ vs. 2 × 10^−2^ day^−1^, respectively). Concomitant immunosuppressive medication, age and differences in immunological mechanisms triggered by the underlying disease may explain the disparities in parameter values [34,40]. Remarkably, the estimates of the magnitude of the inter-individual variability were quite similar to those reported by Pan et al.

Since 2014, different biosimilars of rituximab gained marketing authorization from health authorities worldwide [41]. In this analysis, no effect of the substitution between rituximab drug products could be detected on any model parameters. This represents an interesting finding of the current investigation given the lack of knowledge regarding the interchangeability of these products in children. Still, further studies in larger population settings should be performed to validate the present findings.

Interestingly, the evaluation of the predictive performance of the model in different scenarios such as the administration of rituximab in second o subsequent cycles, provided encouraging results to potentially implement the model during the full treatment period (Figure 2A and Figure 3). Noticeable, predictions resulted especially inaccurate in a patient diagnosed with SLE (see Figure 3, bottom panel). As previously mentioned, the potential effect of ADAs cannot be ruled out since this patient developed an infusion-related reaction in the first rituximab cycle. Moreover, this patient received cyclophosphamide in the first cycle but not in the second. It has been previously reported that there was a synergism between rituximab and cyclophosphamide on B cell depletion [42]. Additional mechanisms could also explain the lack of predictability in this patient during the second cycle [43,44,45,46,47] including differences in immune cells subpopulations with a capacity of rituximab internalization and time-dependent changes in rituximab target-mediated elimination due to dynamic changes in the antigenic targets [38,48].

Despite being widely used, there is a lack of consensus regarding the most optimal therapeutic regimen for rituximab. The most frequently used schemes are 500 or 750 mg/m^2^ every 14 days and 4 weekly doses of 375 mg/m^2^ [3,4]. We used the developed model to explore the impact of the three regimens on CD19+ kinetics. Our results suggest that all of them performed comparatively in terms of CD19+ repopulation times and therefore the selection of the most convenient scheme could be based on patient requirements, tolerability and/or resources availability, without affecting rituximab pharmacological efficacy, in line with previous findings [18]. Also, the number of responders is not significantly affected by the scheme. For example, the regimen could be adapted to reduce hospitalization costs (500 or 750 mg/m^2^ every 14 days vs. 4 weekly doses of 375 mg/m^2^) or to reduce the risk of adverse reactions when lower exposure is preferred (375 mg/m^2^ vs. 500–750 mg/m^2^). These results may be attributed to the restricted range of doses of the evaluated schemes of treatment that are the most commonly used in the clinics. Thus, expanding the dose-range may allow for the detection of other differences not manifested in the current study. Moreover, it is worth mentioning that amounts of CD19+ B lymphocytes in the target organs are not available due to sampling inaccessibility. Therefore, the relation between CD19+ dynamics in the biophase and the clinical response is approximated using CD19+ concentrations in peripheral blood as a surrogate.

In daily practice, difficulties arise in anticipating which patients will respond or not to rituximab [49], mainly due to the absence of predictive biomarkers in neurologic and immune diseases [36,50,51,52]. In the current evaluation, we tried to address that unmet clinical need by searching for a CD19+ related biomarker driving the clinical response. Although our results were not conclusive, patients with either shorter CD19+ B cell repopulation times or higher AUC CD19+ tend to show non-response or stable disease. Of note, we also found no statistical association between clinical response and switching between drug products.

This work has some limitations derived from the relatively reduced sample size and number of observations, which are inherent to paediatric studies, in addition to the lack of PK data and ADAs measurements previously mentioned. The dose-range is also an aspect to take into account in future studies in order to obtain precise estimates. Moreover, a considerable amount of data below the limit of quantification were registered. This is also consistent with depletory therapies targeting B cells. Therefore, studies in larger populations are required to confirm our results.

To the best of our knowledge, rituximab pharmacodynamics studies in neurologic and IHR paediatric patients with off-label use and subjected to biosimilar substitution are not publicly available, thus this study represents novel data in a real-world setting in a large cohort of Latin-American children. Diagnosis, substitution between innovator rituximab and its biosimilars, or type of regimen did not affect rituximab-induced CD19+ depletion nor the clinical response. According to this study, rituximab frequency and dosage may be chosen based on clinical convenience for patient management and considering safety reasons without affecting CD19+ repopulation times. Further studies in larger populations are required to confirm these results. Studies evaluating interchangeability between innovator and biosimilar rituximab in paediatrics are essential to support the decision-making process in daily practice, ensuring comprehensive care with off-label administration of rituximab.

## Figures and Tables

**Table 1:** Main patient and study characteristics.

Training Cohort				Predictive Performance Evaluation Cohort		
Variable	Neurologic	IHR	Total	Neurologic	IHR	Total
No. patients	30	22	52	6	5	11
No. of CD19+ B cell levels	107	88	195	68	18	86
Age (y.o.)	11.1 (0.1–15.4)	11.5 (1.6–18.7)	11.1 (0.1–18.7)	12.1 (7.0–15.2)	13.9 (7.4–17.5)	13.9 (7.0–17.5)
BSA (m^2^)	1.3 (0.2–2.0)	1.2 (0.4–1.8)	1.2 (0.2–2.0)	1.3 (0.9–1.8)	1.4 (0.9–1.7)	1.3 (0.9–1.8)
Body weight (kg)	40 (4–87)	37 (7–75)	38 (4–87)	42 (26–75)	48 (23–75)	43 (23–75)
Sex (Fem/Male) ^a^	18/12	18/4	36/16	5/1	5/0	10/1
CD19+B_0_ (×10^6^cel/L)	437 (5–1953)	315 (5–2513)	544 (5–2513)	83 (34–317)	235 (67–652)	162 (34–652)
Switch between innovator and biosimilar (yes/no/NA) ^a^	8/15/7	6/12/4	14/27/11	0/3/3	0/3/2	0/6/5
Rituximab dose (mg)	500 (41.5–1000)	500 (100–1000)	500 (41.5–1000)	700 (500–1000)	449 (130–1000)	700 (130–1000)
Follow-up time (days)	128 (27–701)	190 (30–872)	199 (27–872)	837 (303–973)	243 (22–688)	688 (22–973)
No. of patients with Co-medication (%)						
Cyclophosphamide	3 (10)	2 (9)	5 (10)	-	-	-
Methotrexate	2 (6.7)	-	2 (4)	-	-	-
Steroids	24 (80)	17 (77.3)	41 (79)	3 (50)	5 (100)	8 (73)
Intravenous immunoglobulin	5 (16.7)	1 (4.5)	6 (11.5)	3 (50)	-	3 (27)

Data are expressed as median (range). ^a^: number of patients. Abbreviations: BSA: body surface area; IHR: immune-haemato-rheumatologic diseases.

**Table 2:** Parameter estimates of the final population pharmacodynamic model (*n* = 52).

Parameter	Estimate	RSE (%)	Shrinkage (%)	Bootstrap ^a^ Median, 95% CI
K_E_ (days^−1^)	0.06	17	-	0.06 (0.049–0.09)
CD19+_0_ (10^6^ cells/L)	353	25	-	352 (248–516)
K_deg_ (days^−1^)	0.004	22	-	0.004 (0.0008–0.005)
EMAX	155	23	-	173 (153–713)
ED50 (mg)	0.692	61	-	0.692 (0.177–2.259)
IIV K_E_ (%) ^b^	55.4	27	47	59.7 (34.8–92.9)
IIV CD19+_0_ (%) ^b^	70.1	32	35	60.5 (8.6–183.0)
IIV K_deg_ (%) ^b^	80.7	43	57	122.2 (39.6–323.3)
Residual error (Ln(10^6^ cells/L))	0.94	9.5	24	0.93 (0.68–1.03)

K_E_: first-order rate constant of removal of rituximab from the blood compartment; K_deg_: first-order rate constant of degradation; E_MAX_: maximum fold increase in K_deg_ that rituximab can elicit; ED_50_: predicted amount of rituximab at any time after its dose administration required to achieve 50% of E_MAX_; IIV: interindividual variability. ^a^: A 95% confidence intervals (CI) obtained from the analysis of 500 bootstrap datasets. ^b^: IIV expressed as coefficient of variation (%) calculated using the expression $\sqrt{e^{\omega^{2}}-1}\times 100$ where ω^2^ is the estimated variance.

## Data Availability

The datasets generated during and/or analysed during the current study are available from the corresponding author upon reasonable request.

## Supplementary Materials

The following supporting information can be downloaded at: https://www.mdpi.com/article/10.3390/pharmaceutics15112534/s1, Figure S1: Simulated CD19+ B lymphocyte individual profiles stratified according to clinical response (*n* = 26); Figure S2: Evaluation of the time of CD19+ repopulation and the area under the CD19+ versus time curve in patients with satisfactory (CR or PR) and non-satisfactory (NR or SD) clinical response; Table S1: Full description of patient diagnosis; Table S2: Demographic characteristics of the subgroup of patients (*n* = 26) with assessment of clinical efficacy; Table S3: Univariate analysis of risk factors for the lack of response to treatment with rituximab (*n* = 26).
