# Supplementary material for: Population Pharmacodynamic Modelling of the CD19+ Suppression Effects of Rituximab in Paediatric Patients with Neurological and Autoimmune Diseases
Source: Pharmaceutics. 2023 Oct 26;15(11):2534. doi: 10.3390/pharmaceutics15112534 (PMC10674351; doi:10.3390/pharmaceutics15112534)
Supplement: Supplementary file 1 [file pharmaceutics-15-02534-s001.zip › pharmaceutics-2609099-supplementary.pdf]

Supplementary materials index

Supplementary figures

Figure S1.

Figure S2.

Supplementary Tables

Table S1. Full description of patient diagnosis

Table S2. Demographic characteristics of the subgroup of patients (n=26) with assessment of clinical efficacy

Table S3. Univariate analysis of risk factors for the lack of response to treatment with rituximab (n=26).

Figure S1. Simulated CD19+ B lymphocyte individual profiles stratified according to clinical response (n=26)

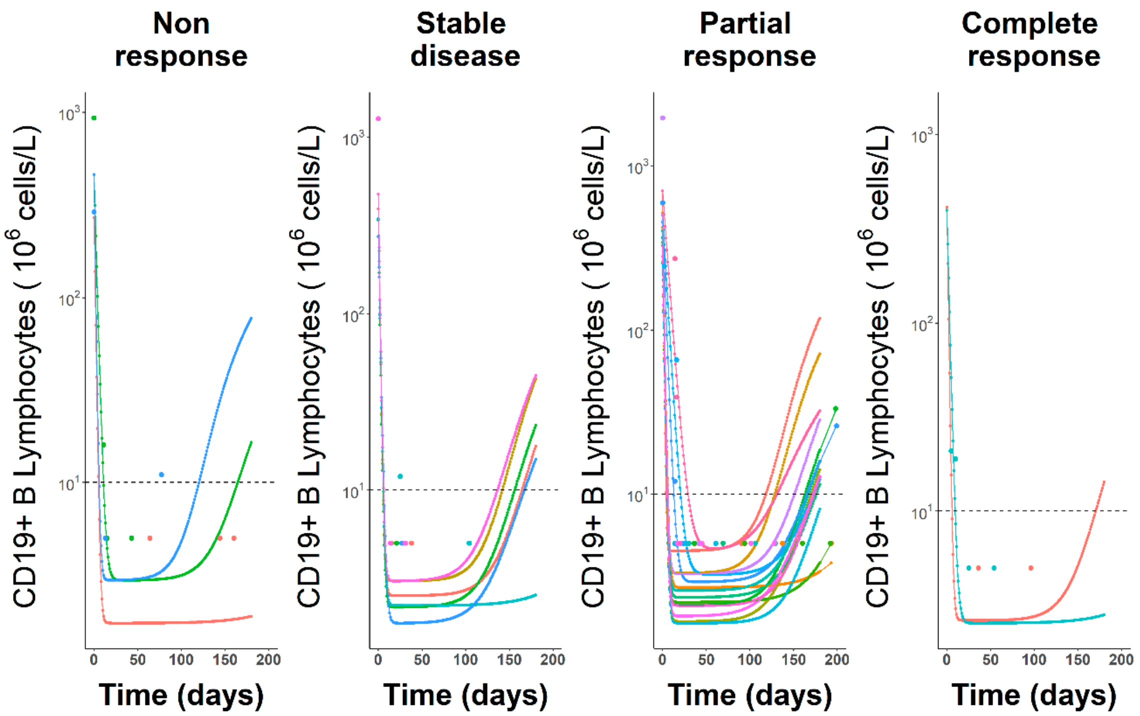

Figure S2. Evaluation of the time of CD19+ repopulation and the area under the CD19+ versus time curve in patients with satisfactory (CR or PR) and non-satisfactory (NR or SD) clinical response

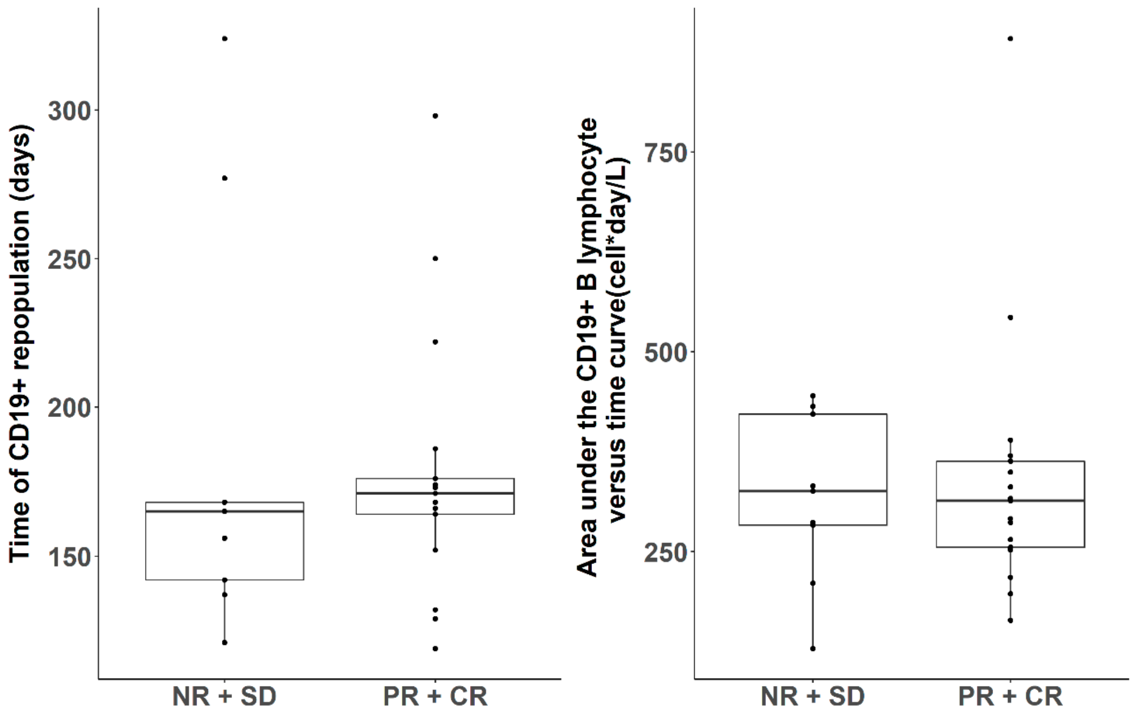

Table S1. Full description of patient diagnosis

**Neurologic Diagnosis**	**Number of patients of the study population (n=52)**	**External predictive performance evaluation cohort (n=11)**
Immune-mediated encephalitis	5	
Multiple Sclerosis	5	1
Neuromyelitis Optica	5	3
ADEM	2	
Pompe disease	2	
Demyelinating disease	2	
CNS inflammatory disease	2	
Seropositive immunomodulated encephalopathy	1	
Immune-mediated epilepsy	1	
Myasthenia Gravis	1	
Myeloradiculitis	1	
Syndrome of Susac	1	
Syndrome Opsoclonus Myoclonus	1	
Chronic ataxia	1	
**Immune-Haemato-rheumatology Diagnosis**		
Systemic lupus erythematosus	11	3
AIHA	3	1
Juvenile dermatomyositis	3	

Pemphigus	1	1
ESJ	1	
ANCA-associated vasculitis	1	
Thrombocytopenia	1	
DiGeorge Syndrome	1	

Abbreviations: ADEM: acute disseminated encephalomyelitis, AIHA: autoimmune haemolytic anemia; ANCA: antineutrophil cytoplasmic antibody, CNS: central nervous system, ESJ: juvenile systemic sclerosis.

Table S2. Demographic characteristics of the subgroup of patients (n=26) with assessment of clinical efficacy

Variable	Neurologic Diagnosis	Immune-Haemato-rheumatology Diagnosis	Total
No. patients	16	10	26
Age (y.o.)	13.0 (1.7-15.4)	12.6 (3.2-18.7)	12.9 (1.7-18.7)
BSA (m^2)	1.3 (0.5-2.0)	1.4 (0.8-1.7)	1.4 (0.5-2.0)
Body weight (kg)	42 (12-87)	46 (21-65)	46 (12-87)
Sex (Fem/Male)	9/7	9/1	18/8
$CD19+_0$ ($x10^6$cel/L)	769 (122-1953)	443 (87-1617)	726 (87-1953)
Switch (yes/no/NA)	3/9/4	2/5/3	5/14/7
AUC_{0-180} (cell*day/L)*	320.7 (128.8-891.7)	288.4 (163.9-542.6)	314.6 (128.8-891.7)
T_{CD19+} (days)	166 (119-324)	170 (121-298)	168 (119-324)
Time of clinical score evaluation (days*)	120 (27-412)	55 (21-111)	85 (21-412)
Clinical response (NR/SD/PR/CR)	1/4/10/1	2/2/5/1	3/6/15/2
No. of patients with Co-medication (%)			
Cyclophosphamide	1 (6.2)	1 (11.1)	2 (7.7)
Methotrexate	0 (0)	0 (0)	0 (0)
Steroids	13 (81)	7 (70)	20 (77)
Intravenous immunoglobulin	1 (6.2)	0 (0)	1 (3.8)

Data are expressed as median (range).

Abbreviations: AUC_{0-180}: area of CD19+B lymphocytes vs time curve at 180 days after initiation of rituximab; BSA: body surface area; CR: complete response; NR: non-response; PR: partial response; SD: stable disease; T_{CD19+}: Time to CD19+ repopulation.

Table S3. Univariate analysis of risk factors for the lack of response to treatment with rituximab (n=26)

Factor	Odds Ratio (IC 95%)	P value
Sex (male vs female)	0.182 (-1.550 – 1.920)	0.84
Diagnose (IHR vs neurologic)	0.383 (-1.260 – 2.030)	0.65
Switch between innovative and biosimilars (yes vs no)	0.061 (-2.380 – 2.500)	0.96
AUC_{0-180} (cell*day/L)	0.002 (-0.003 – 0.007)	0.39
T_{CD19+} (days)	0.002 (-0.013 – 0.018)	0.78
Age at baseline (y.o.)	0.111 (-0.079 – 0.301)	0.25
Female teenagers (yes/no)	0.891 (-1.100 – 2.880)	0.38
BSA at baseline (m^2)	1.146 (-0.690 – 2.980)	0.22
Body weight at baseline (kg)	0.022 (-0.019 – 0.063)	0.29
Absolute lymphocyte counts at baseline (cell/L)	1.945e-05 (-0.0005 – 0.0005)	0.94
Steroids (yes/no)	1.482 (-0.870 – 3.830)	0.22

Abbreviations: AUC_{0-180}: area of CD19+B lymphocytes vs time curve at 180 days after initiation of rituximab; BSA: body surface area; IHR: immune-haemato-rheumatologic diseases, T_{CD19+}: Time to CD19+ repopulation
